# Topical Oestrogen Keratinises The Human Foreskin and May Help Prevent HIV Infection

**DOI:** 10.1371/journal.pone.0002308

**Published:** 2008-06-04

**Authors:** Andrew J. Pask, Kerry J. McInnes, David R. Webb, Roger V. Short

**Affiliations:** 1 Department of Zoology, The University of Melbourne, Melbourne, Victoria, Australia; 2 Prince Henry's Institute of Medical Research, Clayton, Victoria, Australia; 3 Department of Surgery, The University of Melbourne, Melbourne, Victoria, Australia; 4 Austin Health, Heidelberg, Victoria, Australia; 5 The Dean's Ganglion, Faculty of Medicine, Dentistry and Health Sciences, The University of Melbourne, Melbourne, Victoria, Australia; University of Cape Town, South Africa

## Abstract

With the growing incidence of HIV, there is a desperate need to develop simple, cheap and effective new ways of preventing HIV infection. Male circumcision reduces the risk of infection by about 60%, probably because of the removal of the Langerhans cells which are abundant in the inner foreskin and are the primary route by which HIV enters the penis. Langerhans cells form a vital part of the body's natural defence against HIV and only cause infection when they are exposed to high levels of HIV virions. Rather than removing this natural defence mechanism by circumcision, it may be better to enhance it by thickening the layer of keratin overlying the Langerhans cells, thereby reducing the viral load to which they are exposed. We have investigated the ability of topically administered oestrogen to induce keratinization of the epithelium of the inner foreskin. Histochemically, the whole of the foreskin is richly supplied with oestrogen receptors. The epithelium of the inner foreskin, like the vagina, responds within 24 hours to the topical administration of oestriol by keratinization, and the response persists for at least 5 days after the cessation of the treatment. Oestriol, a cheap, readily available natural oestrogen metabolite, rapidly keratinizes the inner foreskin, the site of HIV entry into the penis. This thickening of the overlying protective layer of keratin should reduce the exposure of the underlying Langerhans cells to HIV virions. This simple treatment could become an adjunct or alternative to surgical circumcision for reducing the incidence of HIV infection in men.

## Introduction

In 2006, 39.5 million people were currently infected with HIV, and there were 4.3 million new infections, making HIV one of the greatest health crises in human history. There is a desperate need to develop new methods to prevent HIV infection. The results of three large randomised trials of male circumcision, carried out in South Africa [1], Kenya [2] and Uganda [3], leave no doubt that circumcision more than halves a man's risk of HIV infection [4], [5], and the protective effect is thought to be due to the physical removal of most of the inner foreskin epithelium. This epithelium is richly supplied with Langerhans cells, the main site of HIV entry into the penis [6]. However, Langerhans cells are also a vital part of the body's natural epithelial defence against HIV infection, since they contain the c-type lectin Langerin that normally degrades any virions entering the cell [7], [8]. When large amounts of virus are present, the Langerin reserves may be depleted, so that the Langerhans cells instead become vectors for transporting virus to the regional lymph nodes, establishing a systemic infection.

Langerhans cells are also the primary site of HIV entry into the female reproductive tract [9]. It has long been known that the human vaginal epithelium responds to topical oestrogen administration by thickening and keratinization, and vaginal oestrogen cream or tablets are widely used by postmenopausal women to treat vaginal atrophy [10], [11]. The vaginal epithelial structure has a direct effect on its susceptibility to HIV infection. A decline in oestrogen levels in women after the menopause is associated with a four to eightfold increased risk of contracting HIV [12].

To determine how oestrogen affects the susceptibility of the vaginal epithelium to HIV infection, ovariectomised Rhesus monkeys were treated with either intravaginal oestriol or base cream and then challenged intravaginally with Simian Immunodeficiency Virus (SIV) [13]. Oestriol treated animals showed a dramatically increased resistance to SIV infection; only one of the twelve oestriol treated animals became infected when challenged with SIV, compared to six of the eight ovariectomised controls (p = 0·0044) [13]. The treated animals did become infected if the SIV was injected beneath the vaginal epithelium, showing that the resistance to infection was occurring at the epithelial surface. The increased keratinization of the vaginal epithelium induced by the topical oestriol treatment apparently helped to reduce the number of SIV virions from coming into contact with the Langerhans cells, thus preventing infection.

The inner foreskin is a mucosal epithelium similar to that of the postmenopausal vagina, with a very thin layer of overlying keratin giving little protection to the underling Langerhans cells [6]. We have examined the ability of topical oestrogen to increase keratin production by the inner foreskin epithelium. By thickening the overlying protective layer of keratin, it may be possible to reduce the viral load to which the Langerhans cells are exposed, thus increasing resistance to HIV infection.

## Results

**Figure 1:**
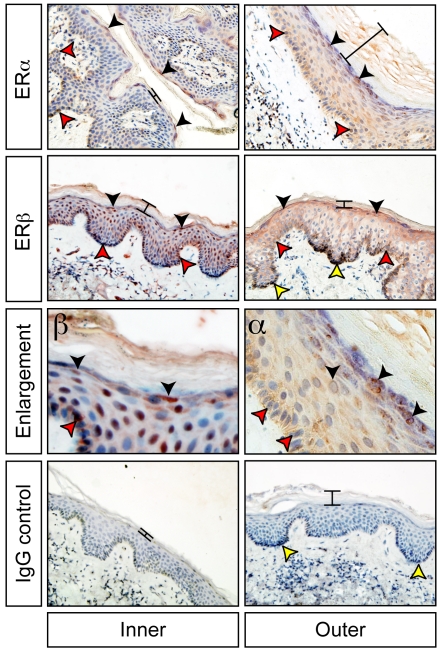
ERα (α) and ERβ (β) protein distribution (brown staining) in the inner and outer aspects of the foreskin. ERα staining was strongest in the stratum granulosum cells (black arrowheads) and in the stratum basale (red arrowheads) with weakest staining in the stratum spinosum. Natural variation in the thickness of the keratin layer is evident between samples (black bars). ERβ shows a similar localisation, but was more prevalent in the dermis than ERα. Negative controls were incubated without primary antibody and with an equivalent amount of non-specific rabbit IgG; as expected there was no staining observed. Melanocytes are present in some sections as histologically dark cells found at the base of the epidermis (yellow arrowheads) and do not indicate staining. Enlarged panels show characteristic cytoplasmic, nuclear and peri-nuclear staining for ERα and ERβ in the epithelium.

In order to determine if the inner foreskin could respond to topical oestriol, we examined the mRNA expression and protein distribution of the two oestrogen receptors (ER) ERα and ERβ, in the foreskins of six adult men aged 30–65, undergoing elective surgical circumcisions. mRNA transcripts for both *ER*α and *ER*β were detected in samples taken from the inner and outer foreskin (Figure S1). ERα and ERβ protein was detected immunohistochemically at high levels in the epidermis of both the inner and outer foreskin of all six men (Figure 1). ERα was predominantly located in the epidermis. Staining was strongest in the stratum granulosum cells and in the stratum basale with weakest staining in the stratum spinosum. ERβ staining was slightly more intense but similar in distribution to ERα. ERβ was more prevalent in the dermis than ERα. These results showed that the epithelial cells of the inner foreskin should be able to respond to topical oestrogen administration.

**Figure 2:**
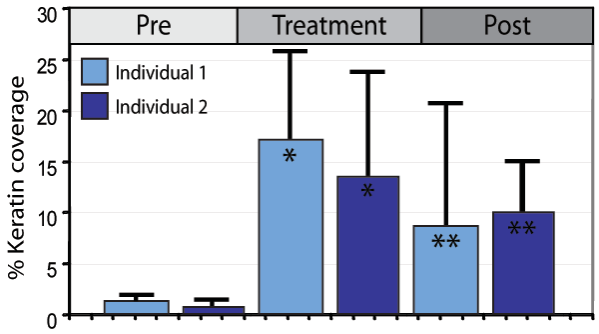
Quantification of desquamated keratin from contact smears. The mean amount of desquamated keratin was calculated for each individual for each treatment period. A highly significant increase in keratin was observed between the 4-day pre-treatment (Pre) and the 14-day treatment periods (Standard T-test; p = 0·002 individual 1 and p = 0·00003 individual 2 indicated by *). A significant increase in keratin was maintained over the 5-day post treatment period (Post) (p = 0·02 for individual 1 and p = 0·01 for individual 2 as indicated by **).

We then examined the ability of oestriol cream to induce keratinization of the inner foreskin of men *in vivo.* Topical oestrogen (Ovestin: Organon, 0.5 ml of cream containing 500 micrograms of oestriol) was applied daily to the inner foreskin of two men. The number of desquamated cornified epithelial cells was recorded in daily contact smears of the inner foreskin (Figure 2, S2) to assess the level of keratinisation. The treatment produced a highly significant increase in the number of desquamated, keratinised epithelial cells within 24 hours of the first oestriol application and this effect persisted for at least five days after the cessation of treatment (Figure 2).

## Discussion

The localisation of both ERα and ERβ in the inner foreskin epithelium, and the increase in keratinization following topical oestrogen treatment, are consistent with the well-documented local effects of oestrogen in the female reproductive tract [9], [10]. Our findings demonstrate that topical oestrogen can induce rapid keratinisation of the inner foreskin. Further studies are now needed to determine if this can protect the Langerhans cells from contact with HIV virions.

In order to avoid any adverse systemic side effects of the topically applied oestrogen, it will be important to determine the minimal effective dose and frequency of application needed to produce the keratinization response. We have chosen to use oestriol since it is a weak, naturally occurring oestrogen metabolite that is normally present in human male urine, and it is unlikely to cause hypothalamic inhibition or gynecomastia. Similar treatment regimens in women [14] had no effect on circulating LH concentrations. A similar keratinisation response was detected in the vaginal epithelium of Rhesus monkeys using as little as 25 µg of oestriol applied twice weekly [13]. Topical oestriol doses as low as these would only induce a local response and would probably be insufficient to cause any changes in circulating oestriol concentrations and systemic side effects.

If the increase in keratinisation of the inner foreskin can prevent HIV entry into the penis, there will be many potential uses for this treatment. Surgical circumcision only confers a 60% protective effect against HIV infection, possibly due to the incomplete removal of the inner foreskin. Perhaps topical oestrogen administration might be able to keratinise the residual inner foreskin tissue, increasing the overall protective effect of circumcision. There is also a significantly increased risk of contracting HIV after circumcision if sexual activity is resumed before sufficient wound healing has occurred [15]. Topical oestrogen has been shown to hasten wound healing [16] and could also be used before and after circumcision to reduce the postoperative wound healing time. Furthermore, whilst male circumcision is undoubtedly effective [17], [18], implementing it is a daunting task, particularly in countries like India, China and most of South-East Asia where it is culturally unacceptable [15]. In such cases, topical oestrogen could provide a medical alternative to circumcision to reduce HIV infection. Oestrogen cream could even be used as a condom lubricant that might ultimately protect the man and the woman.

Thus, this simple, cheap, readily available natural hormone could create a living barrier to HIV that preserves the natural defences of the inner foreskin. The oestrogen treatment could be an invaluable adjunct or alternative to surgical circumcision for reducing the incidence of HIV infection in men.

## Materials and Methods

### RT-PCR

Inner and outer aspects of the foreskin were separated and total RNA was extracted from each separately using the GenElute Total RNA extraction mini kit (Sigma). Extracted RNA was then treated with DNase (DNAfree - Ambion) at 37°C for 30 minutes. RNA was reverse transcribed using a dT primer and the Superscript Reverse Transcription Kit (Invitrogen). PCR primers were designed to span introns; ERα forward 5′ ACTGCATCAGATCCAAGGGAACG 3′ and reverse 5′ GGCAGCTCTCATGTCTCCAGCAGA 3′ amplify an 825-bp ERα fragment and ERβ forward 5′ AGCAGCTGCACTGTGCCGGCAAG 3′ and reverse 5′ CCTCTGCCGGGCTGCACTCGGA 3′ amplify an 839-bp ERβ fragment [19].

### Immunohistochemistry

Tissue sections (8 µm) were treated with 3% hydrogen peroxide in methanol for 15 minutes to quench endogenous peroxidase activity. Antigen retrieval was performed in a microwave on defrost for 20 minutes in 0·5M Sodium citrate pH6·0. Slides were blocked in 10% normal goat serum in phosphate buffered saline. Sections were incubated with primary antibody (NeoMarkers ER Ab-16 and ER-beta Ab-24) at a 1∶400 dilution at 4°C overnight. Antibody binding was detected with goat anti-rabbit biotinylated secondary antibody (Santa Cruz) and amplified using the ABC kit (Vector Labs). Antibody localisation was visualised using 3-amino-9-ethylcarbazole chromogen (red/brown staining, AEC - Vector Labs). Negative controls were incubated with an equivalent amount of non-specific rabbit IgG to primary antibody. Tissues were counterstained with haematoxylin.

### Topical Oestrogen treatment

Two sexually inactive uncircumcised males (74 and 33 years of age) applied 500 µg of oestriol in 0.5 ml of base cream (Ovestin; Organon; 1 mg oestriol/ml) to the preputial sac daily for 14 days. Contact smears were collected daily for four days preceding the treatment, during the treatment, and for six days post-treatment.

### Assessment of Keratinisation response

Contact smears were taken once daily, immediately before application of the oestrogen cream. The foreskin was retracted to expose its inner surface. A poly-L lysine coated glass slide was briefly placed in contact with the exposed inner foreskin surface. Slides were fixed in 70% ethanol, air dried and stored at room temperature until batch staining at the end of the trial. Slides were stained using the Ayoub-Shklar [20] method for visualisation of keratin (shown as brilliant red staining).

## Supporting Information

Figure S1 ERα (top panel) and ERβ (bottom panel) RT-PCR. 1, negative control with template omitted. 2, MCF7 cDNA template (used as a positive control for amplification). 3, inner foreskin sample #1. 4, outer foreskin sample #1. 5, inner foreskin sample #2. 6, outer foreskin sample #2. ERα amplification was performed on all samples; ERβ was performed on sample #2 only. Clear amplification was observed for both receptors in both inner and outer foreskin.
(0.86 MB TIF)

Figure S2 Randomly selected representative images of the stained contact smears in the pre-treatment, treatment and post treatment periods for the two individuals. Keratin stains bright pink. The amount of desquamated keratin increased markedly within 24 hours of beginning the treatment and remained significantly higher than the control period for up to 5 days after cessation of the treatment. Quantification of the desquamated epithelial cells is shown in Figure 2.
(3.80 MB TIF)
