# Supplementary figures and images for: Topical Oestrogen Keratinises The Human Foreskin and May Help Prevent HIV Infection
Source: PLoS One. 2008 Jun 4;3(6):e2308. doi: 10.1371/journal.pone.0002308 (PMC2396280; doi:10.1371/journal.pone.0002308)

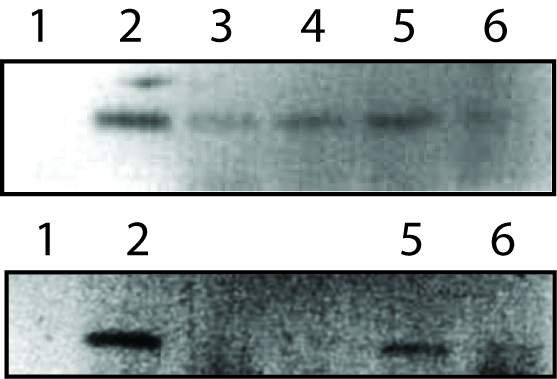

Supplement: Figure S1 — ERα (top panel) and ERβ (bottom panel) RT-PCR. 1, negative control with template omitted. 2, MCF7 cDNA template (used as a positive control for amplification). 3, inner foreskin sample #1. 4, outer foreskin sample #1. 5, inner foreskin sample #2. 6, outer foreskin sample #2. ERα amplification was performed on all samples; ERβ was performed on sample #2 only. Clear amplification was observed for both receptors in both inner and outer foreskin. (0.86 MB TIF) [file pone.0002308.s001.tif]

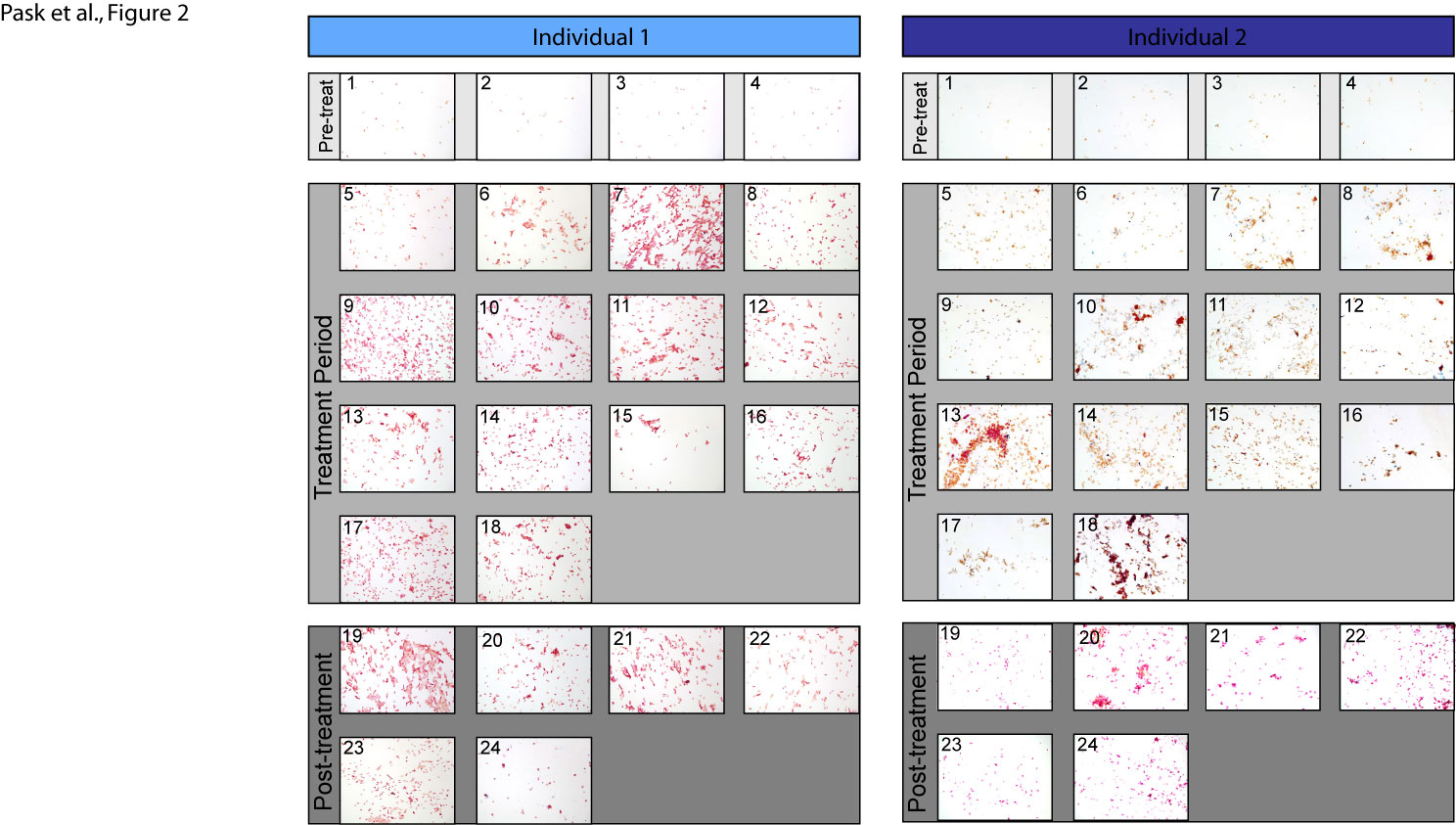

Supplement: Figure S2 — Randomly selected representative images of the stained contact smears in the pre-treatment, treatment and post treatment periods for the two individuals. Keratin stains bright pink. The amount of desquamated keratin increased markedly within 24 hours of beginning the treatment and remained significantly higher than the control period for up to 5 days after cessation of the treatment. Quantification of the desquamated epithelial cells is shown in Figure 2. (3.80 MB TIF) [file pone.0002308.s002.tif]
